# The Heat-Stable Enterotoxin Receptor, Guanylyl Cyclase C, as a Pharmacological Target in Colorectal Cancer Immunotherapy: A Bench-to-Bedside Current Report

**DOI:** 10.3390/toxins9090282

**Published:** 2017-09-15

**Authors:** Trevor R. Baybutt, Allison A. Aka, Adam E. Snook

**Affiliations:** 1 Department of Pharmacology and Experimental Therapeutics, Sidney Kimmel Medical College at Thomas Jefferson University, Philadelphia, PA 19107, USA; Trevor.Baybutt@jefferson.edu (T.R.B.); Allison.Aka@jefferson.edu (A.A.A.); 2 Department of Surgery, Sidney Kimmel Medical College at Thomas Jefferson University, Philadelphia, PA 19107, USA

**Keywords:** colorectal cancer, immunotherapy, vaccine, adenovirus, checkpoint inhibitor, immunotoxin, CAR-T cell

## Abstract

Cancer immunotherapy is becoming a routine treatment modality in the oncology clinic, in spite of the fact that it is a relatively nascent field. The challenge in developing effective immunotherapeutics is the identification of target molecules that promote anti-tumor efficacy across the patient population while sparing healthy tissue from damaging autoimmunity. The intestinally restricted receptor guanylyl cyclase C (GUCY2C) is a target that has been investigated for the treatment of colorectal cancer and numerous animal, and clinical studies have demonstrated both efficacy and safety. Here, we describe the current state of GUCY2C-directed cancer immunotherapy and the future directions of this work.

## 1. Introduction

Colorectal cancer (CRC) is the second leading cause of cancer-related death in the United States, with the 5-year survival prognosis for patients with Stage IV disease being only 11% [1]. Thus, there is an unmet clinical need for novel therapeutics that can better manage, and ameliorate, the substantive burden of late stage disease. In less than a decade, immunotherapy, the practice of employing pharmacological intervention to redirect a patient’s immune system to unleash anti-tumor effector functions, is becoming a primary treatment modality in the oncology clinic. Of most recent success has been monoclonal antibody therapy directed against the T-cell inhibitory signaling molecules cytotoxic T lymphocyte antigen 4 (CTLA-4) and programmed cell death protein 1 (PD-1), collectively referred to as checkpoint inhibitors. These two drug targets have been explored in myriad solid tumors, and the United States Food and Drug Administration (FDA) has recently granted fast track approval for the use of the PD-1 inhibitor nivolumab (Opdivo^®^) for colorectal cancer patients with mismatch repair deficient (dMMR) and microsatellite instability positive (MSI+) disease (CheckMate-142 Trial, NCT02060188) [2]. It should be noted, however, that this therapy is effective in only a subset of MSI+ patients, with a 31% overall response rate across all enrollees (*n* = 74). With MSI+ disease accounting for approximately 15% of sporadic cases, the vast majority of CRC patients, including MSI+ CRC patients unresponsive to nivolumab, still require effective and safe therapeutic options [3].

A major obstacle in cancer immunotherapy has been achieving the optimal balance between specific targeting of tumor-associated antigens (TAA) and broad applicability to the patient population. While checkpoint inhibition has been used in the treatment of melanoma, metastatic renal cell carcinoma, non-small cell lung cancer, and now MSI+ CRC with significant anti-cancer efficacy, the manipulation of such a broad immune regulatory pathway is not without risk. Adverse events arising from the use of checkpoint inhibitors can cause severe autoimmunity across all organ systems, with some cases leading to death [4]. As checkpoint inhibition continues to be used and improved with the development of new checkpoint targets, clinicians are becoming more adept at managing toxicities; however, it is clear that refinement of targeted therapies is required to improve outcomes. The intestinal epithelial, transmembrane cyclase, guanylyl cyclase C (GUCY2C), is an ideal immunotherapeutic target, because it occupies an endogenous, compartmentalized luminal niche protected from systemic autoimmune adverse events, whereas its depolarized expression on metastatic colorectal cancer can be targeted therapeutically.

## 2. Guanylyl Cyclase C

Differential expression of target TAAs has been the solution most exploited for safe and effective immunotherapeutic targeting. The cancer-testis antigens (CTAs), including the family of melanoma-associated antigens (MAGE) and NY-ESO-1, have routinely been used as targets for immunotherapies in multiple cancers due to their ectopic expression in cancer, with limited expression outside testes, which contributes to their immunogenicity and safety profile [5]. Likewise, carcinoembryonic antigen (CEA) has also been used as a target in colorectal cancer; however, the administration of T cells expressing a CEA-specific T cell receptor (TCR) to patients induced severe colitis targeting intestinal CEA, thus limiting its potential [6]. While ectopic antigens aim to exploit temporal differences in expression, cancer mucosa antigens (CMAs) focus on the unique spatial, and environmental, differences experienced by mucosal tissues, where immune function is highly specialized to balance the myriad commensal, food, and environmental antigens encountered regularly with protection from truly dangerous pathogens [7].

**Figure 1:**
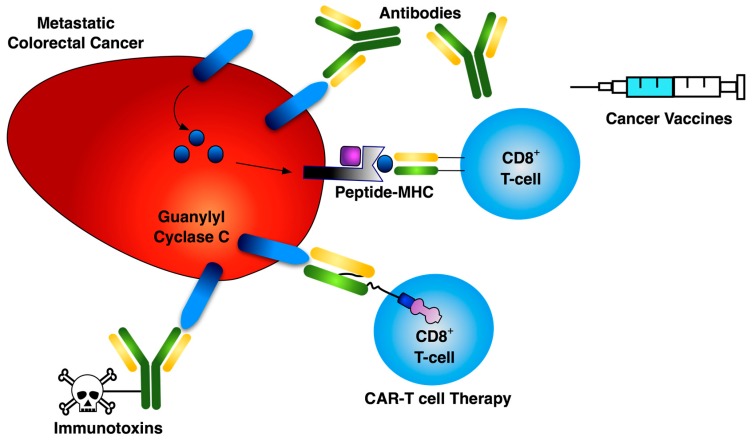
Three Primary Modalities for GUCY2C-directed Cancer Immunotherapy. Immunotherapeutic targeting of GUCY2C encompasses cancer vaccines, immunotoxins, and CAR-T cell therapy, which cover all stages of disease. The primary therapeutic purpose of the vaccine is to provide systemic surveillance against colorectal cancer micrometastases. Immune responses generated by the vaccine are not robust enough to deal with bulky tumor masses. Rather, it primes the immune system to produce GUCY2C-directed CD8^+^ T cell and B cell responses that eliminate micrometastatic disease following standard therapies. GUCY2C-directed CAR-T cells are designed to be highly effective tumor cell killers and the delivery of very large quantities of these cells makes this therapy ideal for bulky disease. However, this autologous therapy is not without risks or high cost and should be reserved for patients with late stage disease unresponsive or refractory to standard therapies. GUCY2C antibody drug conjugates (ADC) are a suitable therapeutic intervention for Stages II, III, and possibly IV, where the tumor has spread and chemotherapeutic standard of care has resulted in an immunocompromised patient where vaccine intervention would be unsuccessful.

This compartmentalization of mucosal immunity supports the therapeutic rationale for targeting GUCY2C [8]. Expressed throughout the lifetime of mammalian organisms, GUCY2C is a transmembrane receptor localized to the apical brush border of the intestinal epithelium, which binds the endogenous ligands guanylin and uroguanylin, catalyzing the conversion of GTP to cGMP, and is required to maintain intestinal homeostasis. GUCY2C is also bound and activated by a heat-stable toxin (ST) produced by enterotoxic *Escherichia coli*, resulting in traveler’s diarrhea [9]. GUCY2C mRNA and protein have also been identified in the salivary gland and hypothalamus, although function is not yet fully understood in these tissues. Importantly, GUCY2C is expressed in greater than 80% of colorectal tumors, with both protein and mRNA expression identified in the majority of primary and metastatic lesions regardless of disease stage or tumor grade [10,11,12]. Moreover, GUCY2C is over-expressed in colorectal tumors, with the apical restriction observed in epithelial cells being lost in malignant cells. This depolarized over-expression, coupled with the immune compartmentalization of the gastrointestinal tract, allows for targeting metastatic tumors enriched in GUCY2C, while the intestine is preserved from adverse autoimmunity [13,14,15,16]. In addition, GUCY2C is expressed in approximately 60% of pancreatic, gastric, and esophageal cancers [17,18,19], suggesting that GUCY2C could be targeted in those diseases as well. To date, GUCY2C biology has been exploited in experimental cancer immunotherapy using vaccines, immunotoxins, and CAR-T cells (Figure 1).

## 3. Vaccines

Once GUCY2C was identified as a target antigen, animal studies were conducted to determine if it could be an effective target and whether autoimmunity would be a limiting factor to the success of this target [20]. Adenovirus, rabies virus, and vaccinia virus vaccines were designed to include the first 430 amino acids of GUCY2C, which encodes its extracellular domain (GUCY2C_ECD_) [20]. BALB/c mice received prophylactic vaccination at 28 days intervals, either receiving a single dose of the adenovirus vaccine, adenovirus plus vaccinia virus, or adenovirus, rabies virus and vaccinia virus, with this heterologous prime-boost strategy being chosen to limit anti-vector immunity when boosting. The CT26 mouse colon carcinoma cell line expressing GUCY2C was implanted subcutaneously and tumor growth kinetics were monitored for 25 days. The adeno + rabies + vaccinia virus vaccination strategy reduced tumor growth and improved survival. In addition, metastatic models including both the liver and lungs were evaluated. In both cases, the tumor burden experienced by animals receiving the GUCY2C vaccine was reduced compared to controls. With vaccine efficacy established in these animal models, concomitant autoimmunity targeting GUCY2C-expressing tissues was examined. Systemic effects of vaccination were determined through measurement of serum chemistries [20]. Creatinine, sodium, triglycerides, and chloride were outside the normal range for both animals receiving either control vaccine or the GUCY2C vaccine, suggesting either a vector- or a strain-specific, but not GUCY2C-specific, effect. Histopathological analyses were conducted for the entire length of the intestine with no evidence of immune cell infiltration or tissue pathology [20]. This work established proof-of-concept for GUCY2C as a safe and effective cancer vaccine target.

The interplay between anti-tumor efficacy and autoimmunity was further examined in animal models of inflammatory bowel disease [21]. After vaccination with GUCY2C-expressing adenovirus (Ad5-GUCY2C), colitis was induced with dextran sodium sulfate (DSS), to investigate if intestinal dysfunction due to inflammatory bowel disease is exacerbated by GUCY2C-specific immunization. There was no difference in body weight or histopathological score between GUCY2C-vaccinated and control-vaccinated animals. Furthermore, GUCY2C vaccination did not increase tumorigenesis in a model of inflammation-associated colorectal cancer. This work demonstrates the potential for safe application of GUCY2C vaccines in a therapeutic setting where CRC patients are presenting with intestinal lesions and possibly intestinal dysfunction.

To improve anti-tumor efficacy in a therapeutic model, adjuvanation of the GUCY2C vaccine with the cytokines GM-CSF or IL-2 was explored [22]. A lung metastases model was established with GUCY2C-expressing CT26 cells, with animals receiving the first vaccination 3 days after tumor implantation, followed by a boost at Day 10. During both vaccinations, GM-CSF or IL-2-expressing plasmid DNAs were administered. There was a significant reduction in the number of lung nodules when animals were sacrificed at Day 15 between animals receiving GUCY2C vaccine and cytokine adjuvanation and those receiving control vaccine and cytokine adjuvanation. The number of lung nodules was no different between control and GUCY2C-vaccinated mice not receiving cytokine adjuvanation, suggesting that the maximal vaccine efficacy in clinical models may be aided by the addition of cytokine therapy. In fact, in both animal models and clinical trials investigating cell-based vaccine therapies for colorectal cancer targeting other tumor antigens, the addition of GM-CSF improved anti-tumor efficacy [23,24,25]. 

In the context of GUCY2C vaccination, cytokine adjuvanation revealed that the maximal therapeutic efficacy of the vaccine had not been achieved by single agent administration. Rather than empirically determining an optimal adjuvant cocktail, the mechanistic determinants impacting immune responses were explored. Investigators observed that anti-cancer efficacy without concomitant autoimmunity was due to lineage-specific induction of CD8^+^ T-cell responses to GUCY2C, without CD4^+^ helper T-cell or antibody responses [21]. This finding suggested that, while vaccination was effective, the opportunity existed to dramatically improve vaccine efficacy if a CD4^+^ helper T-cell response could be induced. The development of GUCY2C-deficient (*Gucy2c^−/−^*) mice permitted the study of self-tolerance mechanisms, which produce selective CD8^+^ T-cell, but not CD4^+^ T- or B-cell, responses to GUCY2C. As expected, *Gucy2c^−/−^* mice immunized with Ad5-GUCY2C, produced robust CD8^+^ T-cell, CD4^+^ T-cell, and B-cell responses, whereas the wild type counterparts did not [26]. Although the mechanisms underlying this selective tolerance remain incompletely defined, it offered a potential strategy to improve vaccine efficacy by engineering a CD4^+^ helper T-cell epitope into the vaccine [26]. The epitope selected was the influenza HA Site 1 epitope, or S1, producing the Ad5-GUCY2C-S1 vaccine [26]. Vaccination of wild-type animals with the Ad5-GUCY2C-S1 vaccine produced GUCY2C-specific antibodies and increased the GUCY2C-specific CD8^+^ T-cell response magnitude [26]. While a GUCY2C-specific CD4^+^ T-cell response was not induced, the S1-specific CD4^+^ T-cell response was sufficient to provide the help necessary for both B and CD8^+^ T cells to improve the GUCY2C response. This improved immune response conferred a substantial improvement in protection against colorectal cancer metastases in lungs, increasing survival 750% compared to the conventional GUCY2C vaccine [26]. Perhaps more importantly to vaccine efficacy than the primary immune response is the ability to generate long-lasting memory responses. The lack of CD4^+^ T-cell help from the conventional vaccine negates the production of GUCY2C-specific CD8^+^ T cell memory responses; however, including the S1 epitope produced memory CD8^+^ T-cell responses, as evidenced by a challenge experiment where Ad5-GUCY2C-S1 vaccinated animals demonstrated a significant survival benefit relative to conventional vaccine recipients following establishment of metastases 8–11 weeks after vaccination [26]. Thus, it was determined that the inclusion of a helper epitope was critical to the clinical success of the GUCY2C vaccine.

As evidenced in earlier work, vaccine efficacy was increased with a prime-boost, rather than prime alone, strategy. However, to overcome immunity targeting viral vectors, a heterologous prime-boost strategy would need to be developed. In the earliest studies, rabies virus and vaccinia virus were used with adenovirus; however, safety concerns with these viral vectors limit their clinical potential. One approach being explored is a DNA vaccine, that would negate the technical challenges posed by vector immunity, and could be used in a heterologous prime-boost strategy with the adenovirus vaccine. Interestingly, a DNA+Ad5 heterologous prime boost strategy safely produced CD8^+^ T cells with substantially higher functional avidity and anti-tumor efficacy than either vaccine alone [27]. 

With the demonstration of both prophylactic and therapeutic efficacy of GUCY2C vaccines, and no observable autoimmunity, the Ad5-GUCY2C-S1 vaccine was developed for clinical use, replacing the CD4^+^ helper T-cell epitope with a universal human CD4^+^ T-cell epitope, known as PADRE [28]. PADRE is a pan DR epitope that binds multiple human HLA DR haplotypes [29]. Starting in 2013, 10 subjects were enrolled in a Phase I clinical trial to determine the safety and immunogenicity of this Ad5-GUCY2C-PADRE vaccine [30]. The study population included patients diagnosed with Stage I or II colorectal cancer and who underwent surgical resection without chemotherapy. The median age was 65 (49–76) with 50% male subjects. Subjects were administered 10^11^ viral particles (vp) of Ad5-GUCY2C-PADRE intramuscularly and then followed for safety and immunomonitoring blood collection at 30, 90, and 180 days following immunization. Adverse events (AEs) were reported by all subjects; however, all AEs were Grade 1 with complaints including expected injection site tenderness and flu-like symptoms. No Grade 3/4 AEs or serious adverse events were observed. Importantly, 4 out of the 10 (40%) subjects produced a GUCY2C-specific CD8^+^ T-cell response. Future Phase II studies may improve responses through repeated Ad5-GUCY2C-PADRE administrations and/or increased vaccine dosing. Further, Ad5-GUCY2C-PADRE may be examined in patients with gastric, esophageal, and pancreatic cancer, which often ectopically express GUCY2C.

## 4. Immunotoxins and Antibody-Drug Conjugates

While vaccines are designed to activate a patient’s immune system to produce an anti-tumor effect, immunotoxins use TAA-targeted antibodies to deliver a toxic payload to TAA-expressing cancer cells. In that context, both mouse and human-specific GUCY2C monoclonal antibodies were developed, and employed as the basis for GUCY2C-targeted immunotoxins. Importantly, these antibodies target the extracellular domain of GUCY2C, but do not bind its ligand-binding site, producing no effect on GUCY2C activation. To produce an effective immunotoxin, the antibody must be internalized to deliver its toxic payload to intracellular compartments. Importantly, upon binding, GUCY2C-specific antibodies are internalized as a receptor-antibody complex via clathrin-mediated endocytosis to endo/lysosomal compartments [31]. The increasing acidification of the endosomal compartment allows for the delivery of a toxic payload conjugated to antibodies via a cleavable bond, disassociating the toxin from the antibody, leading to cell killing through interference of vital cellular processes. For these studies, the ricin A chain toxin was conjugated to a GUCY2C antibody through a cleavable linker [31]. To show therapeutic efficacy, colorectal cancer metastases were established with mouse GUCY2C-expressing CT26 cells and animals were treated with 6 doses every other day beginning two days after tumor challenge. The tumor burden in GUCY2C immunotoxin-treated mice was lower than controls, leading to an increase in survival. Normal tissues evaluated for histopathological signs of damage showed no clinically significant toxicity. These findings have also been translated to a recent Phase I clinical trial examining the GUCY2C-targeted antibody-drug conjugate TAK-264 (MLN0264) in patients with advanced gastrointestinal cancer, producing preliminary evidence of anti-tumor efficacy in some patients, with manageable toxicity [32]. One limitation of immunotoxin or antibody-drug conjugate therapeutics is the development of humoral immune responses targeting the therapy, especially when employing proteinaceous toxins. Indeed, in a clinical trial examining the immunogenicity of a vaccine for ricin A chain, the investigators found a dose-dependent correlation between the vaccine dose and the number of subjects generating neutralizing antibodies (NAbs) [33]. This suggests that identifying less immunogenic toxic payloads or creating multiple toxic conjugates that can be administered sequentially to negate humoral responses to prior treatment may be advantageous. In the context of GUCY2C immunotoxin administration, the impact of NAbs on therapeutic efficacy has not been explored.

## 5. Chimeric Antigen Receptor T Cells

Many immunotherapies leverage the cytotoxic effector function of CD8^+^ T cells to actively kill unwanted tumor cells. T-cell receptors (TCRs) recognize epitopes through the major histocompatibility complex (MHC), which after the T and B cell receptors is the most genetically diverse locus in humans. These TCRs provide the exquisite sensitivity and specificity characteristic of adaptive immune responses. However, effective T-cell therapies are restricted by patient HLA haplotypes, which can be both time-consuming and costly when identifying patient-specific anti-tumor TCRs and their cognate peptide-MHCs. Chimeric antigen receptor (CAR)-T cells circumvent HLA-restriction by combining the endogenous, native protein binding capacity of antibodies with the intracellular signaling domains of TCRs [34,35]. This ability to directly bind the target, rather than a processed peptide epitope, provides flexibility in terms of target, specificity, and broad applicability in the clinic. Using the same antibodies previously described for use with immunotoxins, CAR-T cells were designed by fusing the antibody variable domains into a single fragment (scFv) and linking this to the T-cell activation domains of CD28, 4-1BB, and CD3ζ [36]. This construct was placed in a retroviral vector, which was used to transduce mouse CD8^+^ T cells, making them genetically modified CAR-T cells [36]. Direct binding to GUCY2C by the CAR molecules resulted in T-cell activation and cytokine (IFNγ, TNFα, and MIP-1α) production. Polyfunctional cytokine production (the ability of a single cell to produce more than one cytokine) was observed in over 50% of the CAR-T cell population. In vitro cytotoxicity was measured using plates that report target cell cytotoxicity via electrical impedance (xCelligence, Acea Biosciences, San Diego, CA, USA) and GUCY2C-directed CAR-T cells rapidly kill GUCY2C-expressing target cells (minutes), but did not kill cells lacking GUCY2C. In a model of metastatic colorectal cancer, GUCY2C-directed CAR-T cells reduced the number of tumor nodules and improved survival [36]. Autoimmunity and toxicity were assessed, and there were no apparent signs of tissue damage in normal tissue. CAR-T cells were engineered to express green fluorescent protein (GFP) and GFP^+^ CAR-T cells were observed only in the spleen and tumors, with no evidence of CAR-T cells in the small intestine or colon. Histopathological examination of intestinal tissue showed no signs of pathology attributed to the CAR-T cell therapy. Thus, the GUCY2C-directed CAR-T cell therapy was proven to be both effective and safe in mouse models. To date, GUCY2C-directed CAR-T cell therapy has only been examined in the context of pre-clinical studies. This does not negate the potential for on-target, off-tumor toxicity in the clinic due to differential expression of GUCY2C between mice and humans. As human three-dimensional cell culture matrices are becoming more cost-effective, the ability to examine potential on-target and off-target toxicity prior to direct clinical intervention is increasing in feasibility. It should also be noted that toxicity does not preclude therapeutic usage. CAR-T cell therapy against the B-cell marker CD19 regularly causes B-cell aplasia and cytokine storm in patients, requiring concomitant toxicity management that is both target-specific and outcome-specific [37]. In the context of GUCY2C-directed CAR-T cell therapy, toxicity in the clinical setting can be managed using RNA delivery of the CAR construct, rather than a viral vector, a dose escalation study design, suicide switches, and other approaches [34]. Currently, work is underway to characterize a CAR construct that recognizes human GUCY2C, which can be translated to clinical testing in patients with advanced gastrointestinal cancers.

## 6. Conclusions

Current evidence supports GUCY2C as a safe and effective target for colorectal cancer immunotherapy. With every study, whether it be for vaccines, immunotoxins, or CAR-T cells, an extensive portion of the work has been devoted to examining treatment-induced autoimmunity and toxicity. To date, no treatment modality has demonstrated any signs of serious autoimmunity either in animal models or in clinical trial patients, supporting the hypothesis that anatomical and immunological compartmentalization of GUCY2C prevents intestinal targeting by these therapies. Moreover, both animal models and patient data show that these therapeutics are effective at inducing anti-cancer responses, and suggest that further clinical development of these therapies could greatly improve the lives of patients with GUCY2C-expressing cancers, including esophageal, gastric, pancreatic, and colorectal, establishing GUCY2C-targeted therapies as primary treatment modalities for these deadly diseases.
